# Fat causes necrosis and inflammation in parenchymal cells in human steatotic liver

**DOI:** 10.1007/s00418-021-02030-8

**Published:** 2021-09-15

**Authors:** Eddie Wisse, Filip Braet, Gerald J. Shami, Bartlomiej Zapotoczny, Celien Vreuls, Pauline Verhaegh, Peter Frederik, Peters J. Peters, Steven Olde Damink, Ger Koek

**Affiliations:** 1 Division of Nanoscopy, University of Maastricht Multimodal Molecular Imaging Institute, Maastricht, 6229 The Netherlands; 2 School of Medical Sciences (Discipline of Anatomy and Histology) & Australian Centre for Microscopy & Microanalysis, The University of Sydney, Sydney, NSW 2006 Australia; 3 Institute of Nuclear Physics, Polish Academy of Sciences, 31-342 Krakow, Poland; 4 Department of Pathology, Utrecht University Medical Centre, Utrtecht, The Netherlands; 5 Department of Internal Medicine, Division of Gastroenterology and Hepatology, Maastricht University Medical Center, 6229 HX Maastricht, The Netherlands; 6 Emeritus of Maastricht University, Jekerstraat 39, 6211 NS Maastricht, The Netherlands; 7 Department of Surgery, Maastricht University Medical Center, 6229 HX Maastricht, The Netherlands

**Keywords:** Parenchymal cell, Necrosis, Inflammation, Fat, Human liver, Steatosis

## Abstract

Adapted fixation methods for electron microscopy allowed us to study liver cell fine structure in 217 biopsies of intact human livers over the course of 10 years. The following novel observations and concepts arose: single fat droplets in parenchymal cells can grow to a volume four times larger than the original cell, thereby extremely marginalizing the cytoplasm with all organelles. Necrosis of single parenchymal cells, still containing one huge fat droplet, suggests death by fat in a process of single-cell steatonecrosis. In a later stage of single-cell steatonecrosis, neutrophils and erythrocytes surround the single fat droplet, forming an inflammatory fat follicle indicating the apparent onset of inflammation. Also, fat droplets frequently incorporate masses of filamentous fragments and other material, most probably representing Mallory substance. No other structure or material was found that could possibly represent Mallory bodies. We regularly observe the extrusion of huge fat droplets, traversing the peripheral cytoplasm of parenchymal cells, the Disse space and the endothelium. These fat droplets fill the sinusoid as a sinusoidal lipid embolus. In conclusion, adapted methods of fixation applied to human liver tissue revealed that single, huge fat droplets cause necrosis and inflammation in single parenchymal cells. Fat droplets also collect Mallory substance and give rise to sinusoidal fat emboli. Therefore, degreasing of the liver seems to be an essential therapeutic first step in the self-repairing of non-alcoholic fatty liver disease. This might directly reduce single-cell steatotic necrosis and inflammation as elements in non-alcoholic steatohepatitis progression.

**Supplementary Information:**

The online version contains supplementary material available at 10.1007/s00418-021-02030-8.

## Introduction

Non-alcoholic fatty liver disease (NAFLD) is a worldwide health problem (Friedman et al. 2018), possibly the most important liver disease in the developed world in the twenty-first century (Brenner 2009). Many studies, including a wealth of reviews, confirm the progress of fatty liver disease through the stages of steatosis, necrosis, inflammation, fibrosis, cirrhosis and carcinoma (DeLeve 2015; Friedman et al. 2018). The pathogenesis of many of these sequential stages is not completely understood (Friedman et al. 2018).

Light microscopical (LM) studies of the liver started in the nineteenth century and provided remarkable observations on parenchymal, Kupffer and stellate cells. Starting in the sixties, the application of electron microscopy (EM) contributed to the knowledge of the fine structure and function of the parenchymal cell in normal and diseased liver (Novikoff and Essner 1960; Schaffner et al. 1963a, b; Schaffner and Poper 1963). In both LM and EM studies, immersion fixation was routinely applied. This method preserves the parenchymal cells, but sinusoids collapse, and the fine structure of sinusoidal cells is almost completely lost. Therefore, the early EM studies had difficulties in the distinction between endothelial and Kupffer cells, but concurrently described the space of Disse, the endothelial lining without a basal lamina and the capillarization of sinusoids in steatosis (Ito and Nemoto 1952; Schaffner et al. 1963a; Schaffner and Poper 1963).

However, EM has the potential to study the fine structure of the 20 or so different cell types in the liver (MacParland et al. 2018), provided a type of perfusion fixation is applied. This ensures an instantaneous and simultaneous fixation preserving the shape, topography and ultrastructure of all cells and sinusoids. Owing to this technique, endothelial (Wisse 1972), Kupffer (Wake 1980; Wisse 1974), stellate (Wake 1980) and pit cells (liver resident NK cells) (Kaneda et al. 1983; Wisse et al. 1976) were characterized.

The diagnosis by clinical pathologists using LM is often referred to as a golden standard in the diagnosis, staging and prognosis of liver diseases, including NAFLD (Rockey et al. 2009). Apparently, EM has not succeeded in assisting in the routine microscopic analysis of this disease. Routine methods in both microscopies include the fixation of biopsies, being millimetre-small pieces of tissue, by immersion in a formaldehyde (LM) or glutaraldehyde (EM) solution. Ethanol and other organic solvents, used to process formaldehyde-fixed tissue, dissolve the fat content from the tissues in the LM procedure. This results in the loss of fat, nonetheless one of the key elements in NAFLD. In contrast, the EM procedure involves a minimal exposure to ethanol and uses osmium tetroxide as a second fixative after glutaraldehyde, which uniquely preserves lipid matter. As lipid material is washed out during the preparation for routine LM investigation, no information can be collected about the morphology of the fat droplet and its effect on the liver cells. With our improved method of fixation, lipid material is preserved, which enabled us to investigate the role of fat in NAFLD with a light, scanning and transmission electron microscope.

## Materials and methods

Our tissue samples were collected over a number of years (2009–2019) and can be subdivided into four groups based on different projects or departments (Table 1). The following methods of applying the fixative were used.

**Table 1:** Frequency of occurrence of conditions and structural elements mentioned in the present paper

Quantification of observations per group	Group 1	Group 2	Group 3	Group 4	Total
Number of patients	44	95	21	57	217
Total photos examined	1545	8762	2320	7905	20,532
Steatosis (PA diagnosis and/or much fat on EM)	6	24	13	34	77
Steatohepatitis/NASH (PA diagnosis)	NA	12	0	4	16
Fibrosis (PA diagnosis and/or much collagen on EM)	22	34	13	15	84
Single-cell steatonecrosis (SCS)	1	6	1	2	10
Inflammatory fat follicle (IFF)	1	2	1	1	5
Fat droplets with Mallory substance (FMB)	14	26	0	0	40
Fenestrae normal	11	20	5	17	53
Defenestration	ND	ND	NP	ND	ND
Fat droplet extrusion from parenchymal cells	1	10	7	11	29
Fat embolism in sinusoids	1	10	7	10	28
Inclusions in nuclei of parenchymal cells	10	16	8	23	57
Release of endothelial complex	0	12	2	9	23

Note the frequency of NASH and SCS in group 2, the absence of Mallory substance in groups 3 and 4, and the presence of fenestrae in group 4

*NA* not applicable, *ND* not determined, *NP* normal presence

### Immersion fixation

A percutaneous liver biopsy was taken after local anaesthesia with a cutting-type 16-gauge needle. Parts of biopsies of 1.8–20 mm were cut into 1 mm^3^ blocks and were transferred to a 1.5% glutaraldehyde, 0.067 M cacodylate buffer (pH 7.4) and 1% sucrose (mOsmol 320) for 1 h. After washing in cacodylate buffer, tissue blocks were postfixed in 1% OsO4 with 0.1 M phosphate buffer (pH 7.4) for 1 h, followed by ethanol dehydration and embedding in Epon. LM sections of 2 µm thickness were stained with toluidine blue. EM sections were cut at a thickness of 60 nm and were contrasted with lead and uranyl and studied with a FEI Tecnai G2 Spirit BioTWIN iCorr. Microscopical observations with LM started at low magnifications (4–10×) to supervise larger areas of tissue with a large number of cells. Also, the transmission electron microscopy (TEM) observations started at 48× magnification, allowing to survey about 250 parenchymal cells in one image, prior to the use of higher magnifications up to a maximum of 90,000×. When necessary, a tissue block was further trimmed to select the well-perfused part of the tissue for detailed TEM investigation.

### Jet fixation

Although immersion fixation is commonly applied, it causes fixation gradients due to slow penetration, does not preserve cell shapes well, and causes collapse of sinusoids and and sinusoidal cells. Perfusion fixation provides better results because it fixes all cells in the tissue instantaneously (±30 s) at the cellular level with the same, unchanged fixative. Perfusing a fixative through the portal vein of an experimental liver is relatively easy (Fahimi 1967; Wisse 1970) but cannot be applied to human liver. Therefore, we developed the method of jet fixation (Vreuls et al. 2014).

Needle biopsies were transferred to physiological saline with heparin at 37 °C in a Petri dish, where it was wrapped in gauze. The gauze was gently closed on one side by an artery clamp to arrest the biopsy during spraying by a jet stream of fixative. A bottle with glutaraldehyde fixative, 1.5% in cacodylate buffer 0.067 mol/L, was mounted at a height of 80 cm. The fixative flowed by gravity through a straight tube without obstruction, ending with an 18 G needle. A jet stream of fixative was closely sprayed from a nearly rectangular position onto the biopsy, the needle being gently moved backwards and forwards over its entire length. Spraying time was 2 min with a flow of about 100 mL/min. Halfway through the procedure, the biopsy was turned upside down to spray the other side as well. After spraying, the biopsy was immersed in glutaraldehyde fixative to allow the fixative to fully react with the tissue for a total of 20 min. After this, the tissue was washed in buffer, and further processed as described in the previous paragraph (Wisse et al. 2010). When successful, this method rapidly flushes the tissue until it is fixed, as shown by hardening and a change of colour.

### Injection fixation

By this method, the fixative is injected into a wedge biopsy or a liver lobe (Horn et al. 1987; Wisse et al. 2010). Fixation was successful when the tissue changes colour and hardens during the process, which takes only a few minutes. Depending on the size of the wedge biopsy, the needle can be inserted at different places of the biopsy.

### Patient liver biopsies groups:

#### Group 1 (44 biopsies)

Routine clinical needle biopsies were fixed by immersion.

#### Group 2 (95 biopsies)

Needle biopsies were fixed by jet fixation (Vreuls et al. 2014). We obtained 19 needle biopsies from patients with a diagnosis of NAFLD based upon routine. This group of biopsies contained 12 cases of non-alcoholic steatohepatitis (NASH). The Medical Ethics Committee AzM/UM approved the use of the samples and registered at clinicaltrials.gov NCT02422238.

#### Group 3 (21 biopsies)

Wedge biopsies were fixed by injection fixation (Wisse et al. 2010). Twenty-one wedge biopsy samples from patients with NAFLD were taken between October 2016 and October 2017 in the Zuyderland Medical Center Heerlen, The Netherlands. They were collected mainly during bariatric surgery as part of a project approved by the Medical Ethics Committee Zuyd, registered at clinicaltrials.gov (NCT02717000). All participants gave written informed consent prior to their participation (Verhaegh et al. 2021). The study was performed according to the Declaration of Helsinki (latest amendment of 2013, Fortaleza, Brazil).

#### *Group 4* (57 biopsies)

Wedge biopsies were fixed by injection fixation (Horn et al. 1986; Wisse et al. 2010). Patients undergoing a partial hepatectomy for colorectal liver metastases were included. Biopsies were obtained early after the start of the laparotomy. Of 30 patients, 28 had received oxaliplatin treatment with a median number of 4.5 cycles (range 2–17). The median interval between the last administration of oxaliplatin and surgery was 62.0 days (range 26–1143 days). Wedge biopsies were taken 28–1143 days after the last chemotherapy. The actual number of treatment cycles differed per patient, depending on the radiological response according to Response Criteria in Solid Tumors. In this group of patients, chemotherapy-associated steatosis or steatohepatitis cannot be excluded. Two patients did not receive chemotherapy, but as the other preoperative treatments were similar, these patients served as controls. The total group consisted of 57 wedge biopsies of patients with a history of sinusoidal obstruction syndrome (Vreuls et al. 2012). The study was performed in accordance with the ethical standards of the Declaration of Helsinki, and written informed consent was obtained from each patient.

### 3D array tomography

Array tomography was utilized to generate large cellular volumes at the ultrastructural level as described previously (Moore et al. 2020; Shami et al. 2021). A series of 350 sections (300 nm thick) was generated and collected on a hydrophilized glass slide. Sections were post-stained using uranyl acetate and lead citrate for 10 min each, respectively. The glass slide was carbon coated (5 nm thick) to render it electrically conductive. Inverted backscattered electron imaging was performed using a Zeiss Sigma scanning electron microscope (SEM), operating at 4 kV at a working distance of 5 mm, producing a dataset with the following dimensions: 141 × 143 × 105 µm. Image histogram stack normalization was performed using Fiji (Schindelin et al. 2012), to improve global image contrast. Images were automatically aligned using the StackReg plug-in for Fiji (Thevenaz et al. 1998). Segmentation and visualization was performed via manual tracing using the IMOD software package (Kremer et al. 1996). Volumetric measurements were obtained using the “imod info” script, whilst length measurements were manually obtained using the “measure tool”.

## Results

We studied a total of 217 biopsies of intact human liver, 16 patients were diagnosed with NASH, 77 livers were steatotic and 84 were diagnosed with fibrosis.

### Fixation method

**Fig. 1:**
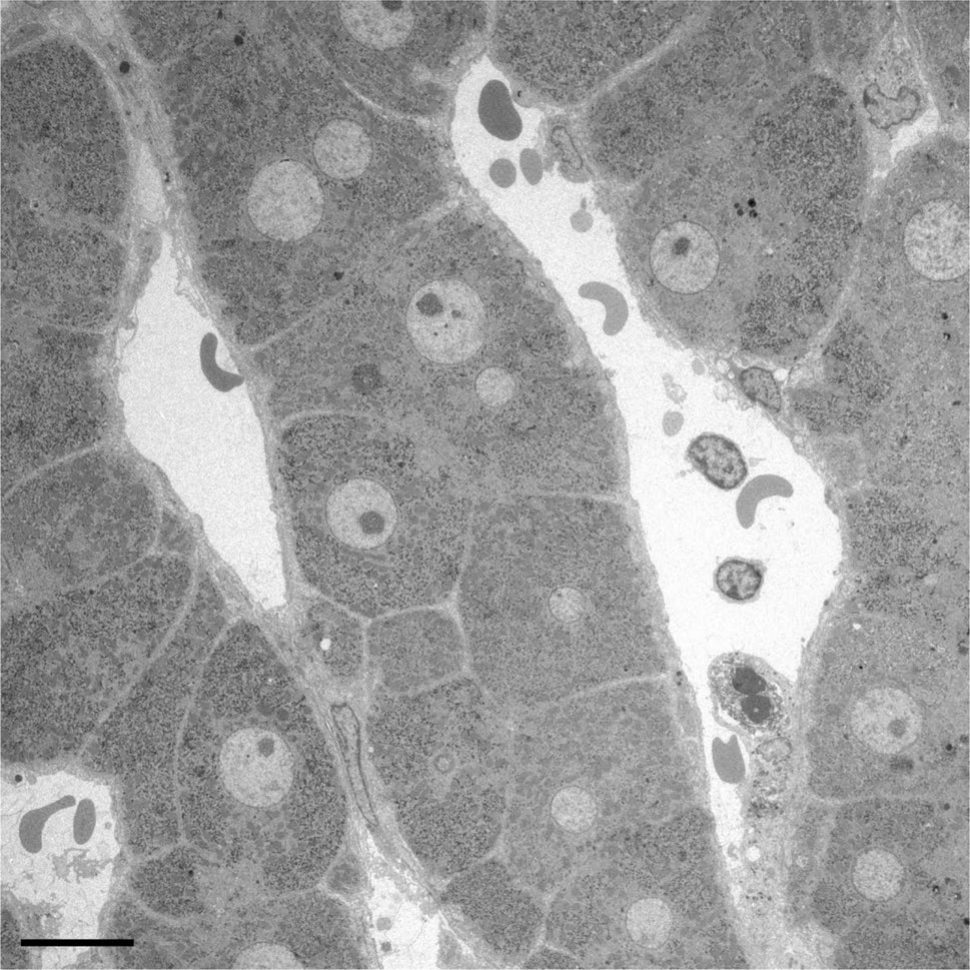
Representative image of perfusion fixation quality of a needle biopsy of diseased human liver. A survey transmission electron microscopy picture of a human liver needle biopsy fixed with jet fixation, of a patient with fibrosis, showing an intralobular region with normal parenchymal cells and sinusoids. Scale bar, 10 µm

**Fig. 2:**
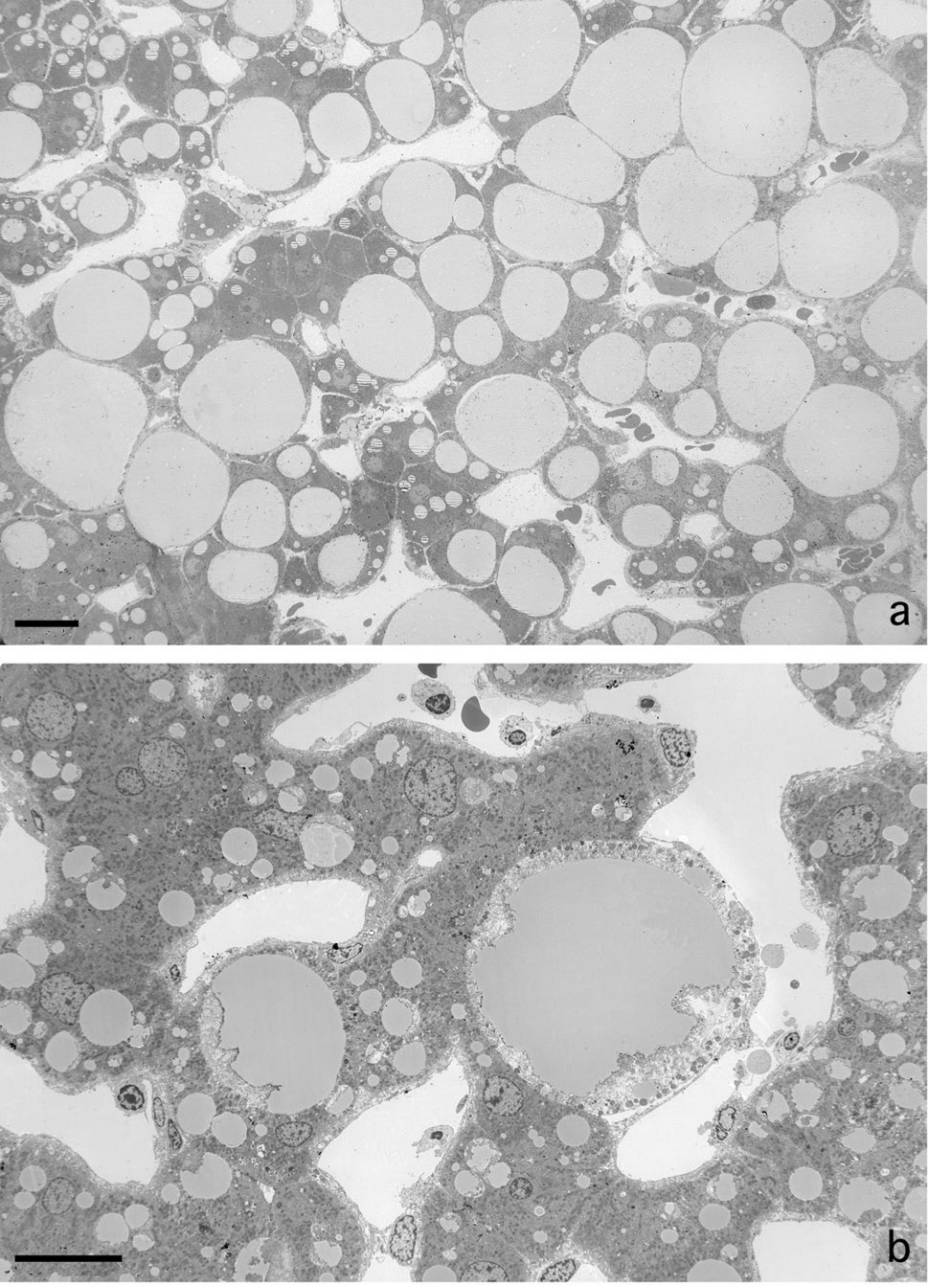
Fat accumulation causes necrosis of liver parenchymal cells at the single cell level. **A** Low magnification of a jet-fixed needle biopsy of a steatotic human liver. Large fat droplets with a preserved lipid content leave little room for the cytoplasms of the parenchymal cells. Notice that these “clean” fat droplets do not contain other material and have an equal electron density. Four stellate cells with small fat droplets, next to the open sinusoids, are dispersed in the tissue. Scale bar, 20 µm. **B** Transmission electron microscopy picture of a single-cell steatonecrosis of a steatotic human liver, in an injection-fixed wedge biopsy. To the right, we observe a parenchymal cell in necrosis with a large fat droplet, an electron lucent cytoplasm, dispersed organelles and morphologically intact plasma membrane. To the left, in a neighbouring cell, a fat droplet with Mallory substance. Scale bar, 20 µm

Improved tissue processing and preservation was obtained by jet and injection fixation, preserving the shape and topography of the cells including detailed cellular fine structure and cellular inclusions such as fat droplets. With immersion fixation, limited observations on parenchymal cells were possible. Regarding jet fixation, the results can be scored as fair to good (Figs. 1 and 2a). Injection fixation of wedge biopsies generally provides good-quality, homogeneous fixation of larger pieces of tissue (Fig. 2b). Essentially, during jet fixation, needle biopsies are sprayed on the outside with glutaraldehyde fixative with the intention to rapidly flush the tissue until it is fixed, as shown by hardening and a change of colour, whereas with injection fixation, the fixative is injected with a needle into a wedge biopsy until the tissue also hardens and discolours. Although both fixation approaches deliver satisfying sample outcomes for subsequent fine structure assessment, we recommend injection fixation as the method of choice when circumstances allow. Over the course of this 8-year-long study, we found that the latter method is quite easy and provides a better score of good results with homogeneous fixation of larger pieces of human liver tissue.

### Presence of fat in parenchymal cells

Livers diagnosed with steatosis show regions of parenchymal cells rich in lipid droplets from small to large (Fig. 2a). These lipid droplets are easily recognizable, because they are round and evenly filled with a material of moderate electron density. Fusion of fat droplets was rarely seen. Lipid droplets do not possess a membrane, as can be seen at higher magnifications (> 40,000×). From our observations we conclude that, sometimes, single fat droplets in a single parenchymal cell grow to such extreme proportions that the droplet marginalizes the cytoplasm, including all organelles, to a narrow peripheral layer. We applied the technique of array tomography, to construct a 3D image of cells in a volume of tissue (Moore et al. 2020; Shami et al. 2021). We measured the mean diameter of parenchymal cells as 29.92 ± 2.54 µm (*n* = 8), the mean volume was 6.01 × 10^3^ ± 2.6 × 10^3^ µm^3^ (*n* = 8). The largest fat droplet had a diameter of 38.99 µm and a volume of 24.29 × 10 µm^3^ or four times the volume of an average parenchymal cell without fat.

### Single cell steatonecrosis

Solitary necrotic cells, still with one huge fat droplet inside, are testimony of a process that can be denominated as single-cell steatonecrosis (Fig. 2b). Although the general fine structure of the cell is disturbed, organelles can still be recognized while dispersed in an electron-lucent, disorganized cytoplasm. A cell membrane is still present and draws the contours of the dead cell. In addition, the endothelial lining is intact and avoids the possibility of organelles escaping and entering the blood stream. In this case, necrosis is a peculiar single-cell process and does not include the usual large number of neighbouring cells as is often the case in tissue necrosis.

### Inflammatory fat follicle

**Fig. 3:**
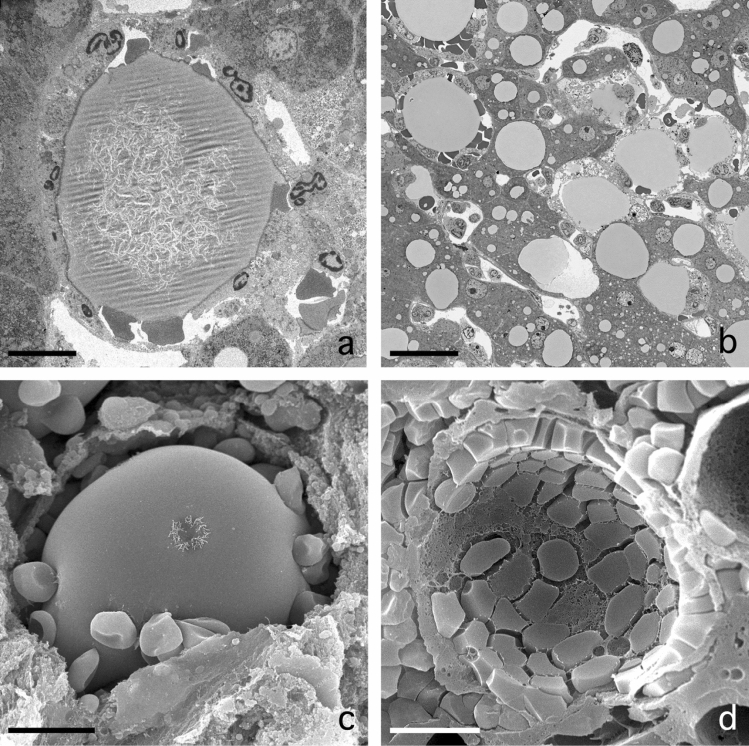
Inflammation at the single cell level. **A** Transmission electron microscopy picture of an inflammatory fat follicle in a human liver fixed with injection fixation, showing a fat droplet surrounded by inflammatory cells and a few red blood cells. Scale bar, 10 µm. **B** One of a sequential series of 350 micrographs together composing a three-dimensional dataset, observable as a video through the following link: https://www.dropbox.com/s/ho2l4cju16gj1vn/HM53%20Z-stack.avi?dl=0. The figure contains two SCNs, four inflammatory fat follicles, and large fat droplets; in the top left corner, an inflammatory fat follicle, showing neutrophils and erythrocytes surrounding a large fat droplet in a necrotic parenchymal cell; in the middle of the picture, a single-cell steatonecrosis, showing a large fat droplet not yet surrounded by neutrophils. Three other inflammatory fat follicles are visible at 12, 14 and 17 o’clock. Neutrophils are also seen in the sinusoids. The three-dimensional video is not based on a timeline, but takes you on a trip along the *Z*-axis down the tissue block. The preparation regards an injection-fixed wedge biopsy of a steatotic human liver with inflammation. Scale bar, 30 µm. **C** Scanning electron microscopy (SEM) picture of an inflammatory fat follicle, showing the uptake, or release, of filaments, supposedly being Mallory substance. As in Fig. 3A, the fat droplet is surrounded also by red blood cells. Scale bar, 10 µm. **D** SEM image of an inflammatory fat follicle. During preparation, the fat droplet has dropped out, leaving behind the cells that directly surrounded the fat droplet. The cells involved are neutrophils and red blood cells, apparently forming a monolayer of closely neighbouring cells. Neutrophils show a different contact pattern as compared with the smooth surface of the red blood cells. Scale bar, 10 µm

As a sequel to this single-cell steatonecrosis situation, we observe necrotic cells where the single fat droplet is surrounded by a layer of neutrophils and red blood cells (Fig. 3a–d). Phagocytosis of fat by the neutrophils was not seen. We propose to name this phenomenon inflammatory fat follicle, explaining the presence of a monolayer of inflammatory neutrophils surrounding a remaining fat droplet. Notice that neutrophils, which are normally adherent to the wall of the sinusoids, should have crossed the endothelium, the space of Disse and the parenchymal cell membrane to adhere to the fat droplet. The number of neutrophils per fat droplets seems to be quite large as seen from a 3D point of view (Fig. 3a–d).

### Sinusoidal lipid embolus

**Fig. 4:**
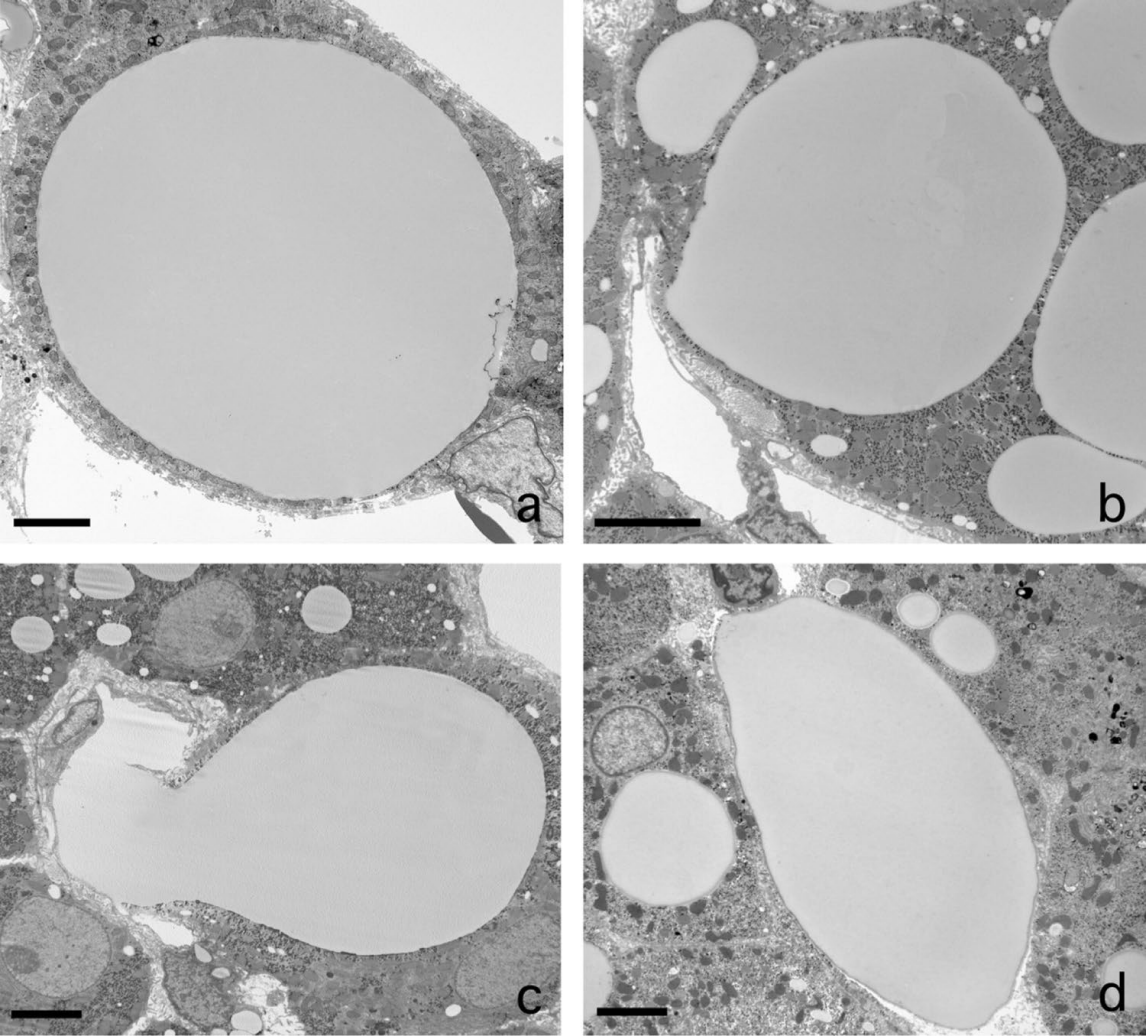
Escape of fat from parenchymal cells. **A** Transmission electron microscopy picture of a huge fat droplet in a parenchymal cell of a patient with liver steatosis. The fat droplet is free of filaments and Mallory-like substances, except for a little dense structure at the right margin of the droplet. To the left and the top right are the lumina of two sinusoids. At the bottom right we observe a sinusoidal endothelial cell. **B** Formation of a nozzle pointing into the direction of the sinusoidal membrane of the parenchymal cell. **C** Extrusion of a fat droplet, breaking out of the parenchymal cell, traversing the space of Disse and the endothelial lining and entering the lumen of the sinusoid. **D** Sinusoidal fat embolus: a large fat droplet is plugging the lumen of a sinusoid. Scale bars, 5 µm

A rather frequent observation regarding fat is the escape of single large fat droplet from a parenchymal cell (Fig. 4a–d). Although fat droplets are round, sometimes a kind of nozzle forms that points into the direction of the sinusoid. The fat droplet narrows the cytoplasm separating it from the sinusoidal membrane of the parenchymal cell. The next step is the disruption of the thin layer of cytoplasm and the escape of the fat droplet into the space of Disse, followed by traversing the endothelium. Next, the fat droplet adapts to the shape of the sinusoid and blocks the sinusoidal blood flow, and a sinusoidal lipid embolus is born. Remarkably, there is no attachment of neutrophils to these sinusoidal fat droplets, in contrast to what we observe in an inflammatory fat follicle.

### Mallory substance in fat droplet

**Fig. 5:**
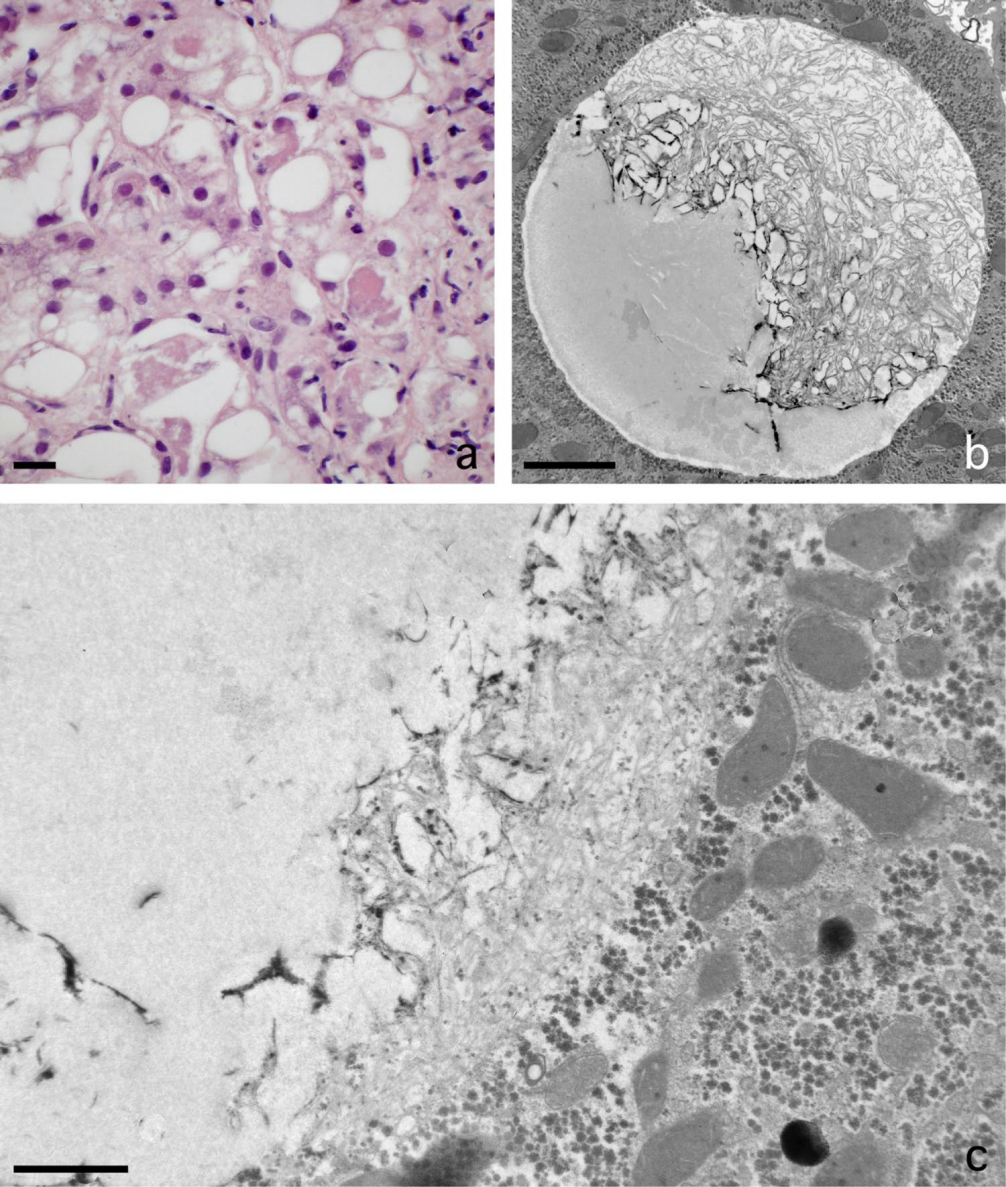
Mallory bodies (MB) developing within fat droplets. **A** Light microscopical picture of a pathological routine paraffin section showing eosinophilic MB in the lower half of the figure. The darker, irregular structures are the MB. Scale bar, 20 µm. **B** Transmission electron microscope (TEM) image showing a lipid droplet of a jet-fixed needle biopsy of a patient with non-alcoholic steatohepatitis. The fat droplet is more than half filled with a mass of filamentous and irregular material, shown at higher magnification in Fig. 5C. In the same liver, we find droplets with less Mallory substance. Scale bar, 2 µm. **C** TEM picture with higher magnification of the material included into the fat droplet in the liver of a non-alcoholic steatohepatitis patient. Pieces of filaments are clearly present, next to irregular material that is difficult to describe. Note that there is no membrane between the lipid droplet and the cytoplasm of the parenchymal cell. Scale bar, 1 µm

In several biopsies, the lipid droplets in parenchymal cells contain clustered fragmentary filaments mixed with masses of other membranous and irregular material (Fig. 5a–c). This material is located at the periphery of the fat droplets but is always confined within the rounded contour of the fat droplet. The quantity of this material varies from nothing to intense. It is supposed that this stored material is formed in the cytoplasm, is lipophilic and has accumulated in the membrane-less fat droplet. The material included in the fat droplets can be considered equivalent to the material of Mallory bodies (MB) as observed in LM preparations (Denk et al. 1979; Zatloukal et al. 2007) (Fig. 5a). No other observed organelle, structure or material could possibly represent the content of MB in the parenchymal cells of our 217 biopsies. Therefore, we conclude that the described structure most probably represents MB integrated with the fat component, and as such these could be called fatty MB. No comparable material free from fat was found elsewhere in the cytoplasm of parenchymal cells. Fatty MB were most frequent (9/12) in patients with non-NASH in group 2. Remarkably, in groups 3 (NAFLD) and 4 (normal liver next to metastatic tissue) with a total of 79 patients, fatty MB were completely absent.

### Endothelial fenestrae and fat

**Fig. 6:**
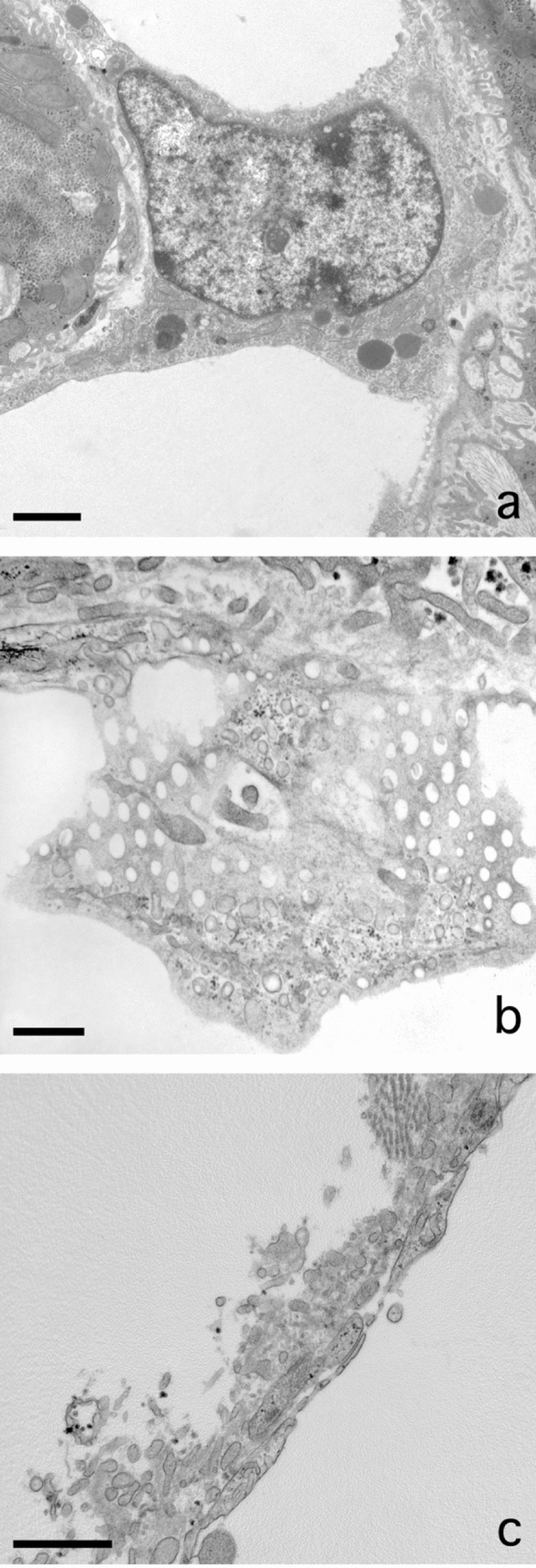
Liver sinusoidal endothelial cells and fenestrae in NASH. **A** Transmission electron microscope (TEM) picture of a sinusoidal endothelial cell in a jet-fixed needle biopsy of human liver of a patient with non-alcoholic steatohepatitis (NASH). Note the presence of organelles such as mitochondria, lysosomes (dense bodies), a nucleus with eu- and heterochromatin and a nucleolus, many pinocytotic vesicles and in the right lower corner the connection with a sieve plate. Scale bar, 2 µm. **B** TEM image of fenestrae arranged in a sieve plate in a sinusoidal endothelial cell in a jet-fixed needle biopsy of an intact human liver of a patient with NASH, illustrating that normal fenestrae are present. This liver and other livers with NASH, contain a normal population of fenestrae as the picture is showing. To image sieve plates, it is necessary that the thin layer of endothelium is within the volume of the ultrathin section and has an orientation parallel to the surface of the section. Scale bar, 0.5 µm. **C**: TEM picture showing an “endothelial complex” ruptured off from the surface of the parenchymal cell. The complex consists of the endothelial lining, with recognizable fenestrae, processes of stellate cells, microvilli of the parenchymal cell and fibres and fluffy material belonging to the extracellular matrix normally present in the space of Disse. It is supposed that this complex has been ripped off as a coherent unit by the force of the fluid stream during injection fixation. The patient had received chemotherapy for colon carcinoma metastasis, last chemotherapy 132 days before the wedge biopsy was taken. The liver was diagnosed as steatosis scale 3. Scale bar, 1 µm

Endothelial cells were present in all preparations displaying normal morphology. The presence of fenestrae was also used as an additional structural marker for tissue preservation. Our attempt to measure fenestrae in group 3 livers failed because of their absence or lack of statistically sufficient numbers of fenestrae. Naturally, this indicates defenestration that was apparently present in these livers. Importantly, fenestrae were again present in NASH livers (Verhaegh et al. 2021) (Fig. 6a, b). We were able to measure 1267 fenestrae in the livers of five NASH patients, the average diameter being 123.5 nm. In group 4, we measured the diameter of fenestrae in ten wedge biopsies to be 102.9 nm (±1.69, *n* = 2811), which is slightly below the normal average value of 107 nm for human liver fenestrae (Wisse et al. 2008).

### Endothelial complex

In some cases, with jet or injection fixation, the endothelial wall of the sinusoid detached locally from the underlying parenchymal cell (Fig. 6c). Amazingly, fragments of stellate cell processes, microvilli of parenchymal cells, extracellular matrix and collagen fibres remained attached to the endothelium after being ripped off the parenchymal cell as a coherent endothelial complex. This suggests a strong coherence of the mentioned components. The detachment was probably due to a strong flow of fixative during jet fixation, resulting in the focal rupture.

## Discussion

This study investigated the role of fat droplets in the livers of patients with different stages of NAFLD at the ultrastructural level. With an improved method of fixation, it was possible to observe specific patterns of fat droplets in liver parenchymal that could not be studied with LM and classical fixation. The instantaneous and simultaneous perfusion type of fixation preserved not only a number of fat-driven cellular processes, but also preserved all cell types, sinusoids and topography. Importantly, original observations were the phenomena defined as “single-cell steatonecrosis”, “inflammatory fat follicle”, “sinusoidal lipid emboli” and “fatty Mallory bodies”.

The capacity of parenchymal cells to store fat and glycogen is enormous. During steatosis, part of the parenchymal cells is filled with fat droplets of different sizes. The change in size of the steatotic parenchymal cells has an inhibitory effect on the microcirculation, as observed by in vivo microscopy in mice with experimental steatohepatitis (McCuskey et al. 2004).

In steatosis, most parenchymal cells contain only one large fat droplet with a volume many times larger than the original cell. Such a large fat droplet marginalizes the cytoplasm and the organelles, which will probably disturb the metabolism and transport processes within the cell. Because we observed single necrotic cells still containing such a large fat droplet, we postulated that in such an instance the cell has been killed by its own huge fat droplet. Apparently, a cellular mechanism to stop excess fat accumulation is absent. Stereological hindrance, disruption of cytoplasmic compartments and difficulties with intracellular transport seem to be candidate determining factors. When a point of no return is reached at which the cell’s metabolism is fatally disturbed, the cell dies. Large fat droplets having different sizes might explain why the killing process is a dispersed, single-cell process and not a regional process at the tissue level.

Single-cell steatonecrosis is a typical necrosis-type of cell death different from apoptosis. After its original definition (Kerr et al. 1972), apoptosis is a morphologically well-defined and easily recognizable process. Remarkably, in all 217 biopsies in this study, we did not observe a single case of apoptosis in any of the 21 hepatic cell types (MacParland et al. 2018), Normally, necrosis is a tissue affair, and takes place simultaneously with a large number of neighbouring cells, while being caused by an interruption of the (micro) circulation or by a low oxygen level. Here we have an example of a single cell death with the typical characteristics of necrosis, showing a disintegrated cytoplasm, a leaky cell membrane leading to a loss of electron density of the cytoplasm (as an expression of the loss of cell mass). We assume that single-cell steatonecrosis is a specific type of hepatic injury contributing to the transition of steatosis to steatohepatitis.

We suppose that single-cell steatonecrosis is a structural process more precise than ballooning as observed by LM. We understand that ballooning refers to a large empty space left after a large fat droplet has been dissolved away by ethanol (Caldwell et al. 2010). Ballooning is an important hallmark of NASH (Friedman et al. 2018) and might correlate with the number of single-cell steatonecroses. The two phenomena are different expressions in the same process visualized by different microcopies, although ballooning might also occur when necrosis has not yet set in. Ballooning has also been reported to correlate with the presence of MB (Zatloukal et al. 2007).

We observed a subset of single-cell steatonecrosis showing the presence of neutrophils and red blood cells surrounding a fat droplet. These invading cells were closely adherent to the fat droplet and often constitute a monolayer. Organelles and parts of the cell membrane of the original parenchymal cell were still present. We consider the neutrophils in this situation to be executing a genuine inflammatory action, because they extravasated the sinusoid by passing through the endothelial lining, crossing the space of Disse, passing the parenchymal cell membrane, and finding their way to the fat droplet, apparently considering it to be a “foreign” body to be attacked. We suspect that the red blood cells present have entered the scene more or less by accident. They probably squeeze in while the neutrophils forced their way to the necrotic cell. Remember that red blood cells in the circulation outnumber white blood cells by a factor of thousand. Therefore, their presence might be considered a kind of “contamination” because there does not seem to be a biological meaning for their presence. Neutrophils are also present in a normal sinusoid where they adhere to the luminal side of the endothelium as a quiescent, surveying population. This does not exclude that this population of neutrophils is also responding to a molecular crosstalk and cooperate with their active family members within the inflammatory fat follicles. We expect the inflammatory fat follicles to initiate or contribute to the early stages of steatohepatitis.

Among the 217 livers studied, 77 were diagnosed with steatosis, either by a pathologist or when much fat was seen on EM. In 10 livers we found single-cell steatonecrosis, 5 livers contained inflammatory fat follicles, and 16 livers were diagnosed with NASH and 84 with fibrosis (Table 1). In sections of livers with inflammatory fat follicles, we saw one to ten inflammatory fat follicles at different locations within one EM section. Considering these relative low numbers, one could ask whether inflammatory fat follicles can be responsible for causing inflammation of the liver in steatohepatitis. The fat droplet in an inflammatory fat follicle is large, and 3D-wise it hosts many neutrophils that, together with all other inflammatory fat follicles, can build a vigorous army involved in the process of inflammation. Inflammatory fat follicles have a characteristic morphology; we conclude they represent a specific, peculiar stage in the development of steatohepatitis. A good impression of the occurrence of single-cell steatonecrosis and inflammatory fat follicles is given by observing the video of a liver 3D tissue block made by array tomography (Moore et al. 2020; Shami et al. 2021) (Fig. 3b).

As a result of these observations, fat can be put in the spotlight for being responsible for triggering two subsequent stages in NAFLD: first, mechanically compromising cell function by accumulation causing necrosis and, second, triggering inflammation.

Mallory bodies (MB) described by Mallory (Mallory 1911) have been considered indicative of alcoholic liver disease and other chronic liver diseases, but their diagnostic value has apparently been diminished. MB contain fragments of intermediary filament keratin (Franke et al. 1979) and are the result of misfolding of cytoskeletal proteins and proteasome overload. MB on routine LM show a very irregular, eosinophilic mass in an empty space in the parenchymal cell. Unfortunately, EM descriptions of MB in situ are very rare. MBs are often observed in livers showing ballooning and fibrosis. MB do not possess a limiting membrane and contain not only filamentous structures but also material difficult to describe (Denk et al. 1979; Franke et al. 1979; Zatloukal et al. 2007).

We have been extensively searching for MB in our collection of biopsies but were unable to find a specific organelle or structural mass that could possibly be considered to be a MB. Otherwise, it became clear that lipid droplets in parenchymal cells often contained variable amounts of fragmented filaments and other material, confined within the limits of the fat droplet. We consider the possibility that these filament-containing fat droplets are the EM representation of MB. Regarding our method of fixation, the clue could simply be the preservation of fat integrated with Mallory substance as fatty MB. We found them to be present in 40 out of 139 biopsies in groups 1 and 2. Remarkably, the fatty MB are completely absent in the 78 patients of group 3 and 4. This absence of MB raises the question of what underlying stimulus responsible for forming MB is missing in these groups.

The application of higher EM magnifications, however, lowers the detection limit for the presence and the quantitation of the Mallory substances included in the fat droplet. A few short filaments can be observed easily on EM at higher magnification (> 40,000×), but they will most probably escape LM observation even with immunocytochemical methods.

The phenomenon of large fat droplets escaping from the hepatocytes causes fat embolies in the sinusoid and is a candidate for the blocking of sinusoidal blood flow quite frequently observed in our preparations (Table 1). Forces exerted on the growing fat droplet might push it towards the sinusoidal side of the cell where a thin rim of cytoplasm is ruptured and the fat droplet escapes into the space of Disse on its way to the sinusoid. Once arrived in the sinusoid, the intact lipid droplet, still intact, will settle as a sinusoidal embolus. In addition, we observed neither large empty holes in parenchymal cells nor ruptured cytoplasm or holes in the endothelial lining. This could mean that the fat droplets escape from living cells which restore their structure after the fat discharge.

Sinusoidal endothelial cell fenestrae (Arias 1990; Wisse 1970; Wisse et al. 1985) are a central theme in many studies concerning NAFLD livers. They are play a role filtering the bidirectional transport of several kinds of lipoproteins by their size, thereby influencing the cholesterol uptake and its synthesis by parenchymal cells (Fraser et al. 1995). Fenestrae in human liver were found to have an average diameter of 107 nm (Wisse et al. 2008), while other species, such as mice and rats, have rather larger diameters of 145 nm as measured in ultrathin sections with transmission electron microscopy. Critical proof of filtration was found in neonatal rats (Naito and Wisse 1978), and was also given by the comparison of different fenestrae diameters in rabbit and mouse livers, where only the larger fenestrae let an adenoviral vector pass (Wisse et al. 2008). Reported defenestration in steatotic livers results in the creation of capillaries with more or less continuous endothelium (DeLeve 2015; Horn et al. 1987; Schaffner et al. 1963a). Importantly, defenestration occurs in early phases in NAFLD patients (Verhaegh et al. 2021). However, according to our present observations, fenestrae return in NASH (Fig. 6B). In total, we measured 1267 fenestrae in five NASH livers that showed an enlargement of fenestrae diameter from 107 to 123 nm. An explanation for the defenestration and refenestration is to date not available.

Apparently, fenestrae are dynamic structures in short time experiments and maintain their shape or change their diameter in cooperation with the fenestrae-associated cytoskeletal ring (Braet et al. 1995). As studied by atomic force microscopy on living endothelial cells in culture (Zapotoczny et al. 2019), fenestrae move within sieve plates, open and close within an average time frame of 20 min, and do not need a fenestrae forming centre (Braet et al. 1998) to open. Fenestrae are part of a dynamic process that is not yet clearly understood in the pathophysiology of NAFLD.

Focal detachment of the endothelium in combination with a firmly attached extracellular matrix, stellate cell processes and disrupted microvilli of the parenchymal cell was observed. The images suggest a strong cohesion of the mentioned cell components (Wake 1980) apparently forming a physically coherent endothelial complex. The reason for loosening is most probably the force of the flow during injection of the fixative. The described construction indicates a strong cohesion of the mentioned elements, also present in normal, intact conditions.

## Conclusions

This rigorous observational study of over 200 human liver biopsies showed for the first time that, with EM and new standardized fixation methods, extensive accumulation of fat in single fat droplets caused a special form of liver injury in the form of single parenchymal cell steatonecrosis, followed by inflammatory fat follicles and sinusoidal lipid emboli. The combination of fat and Mallory substance appeared in our EM observations that is not seen in LM. Apparently, degreasing of the liver is an essential therapeutic first step in the prevention of the progression to non-alcoholic steatohepatitis by reducing lipid toxicity. The role of EM can be of help in further discovering these pathophysiological cellular mechanisms related to NALFD and could be used in future research.

## Supplementary Information

Below is the link to the electronic supplementary material.

Supplementary file1 (DOCX 101757 KB)

## Data Availability

The datasets generated and/or analysed during the current study are available from the corresponding author on reasonable request.
