# Supplementary material for: Fat causes necrosis and inflammation in parenchymal cells in human steatotic liver
Source: Histochem Cell Biol. 2021 Sep 15;157(1):27–38. doi: 10.1007/s00418-021-02030-8 (PMC8755686; doi:10.1007/s00418-021-02030-8)
Supplement: Supplementary file 1 [file 418_2021_2030_MOESM1_ESM.docx]

Wisse et al_HACB-D-21-00198
